# The Necessity of an Ambulance System in Chicago

**Published:** 1884-04

**Authors:** G. Frank Lydston

**Affiliations:** Late Resident Surgeon Charity Hospital, and State Emigration Refuge and Hospital, New York City; 125 State St.


### ARTICLE VII.

### THE NECESSITY FOR AN AMBULANCE SYSTEM IN CHICAGO. By G. FRANK LYDSTON, M.D., late Resident Surgeon Charity Hospital, and State Emigration Refuge and Hospital, New York City.

To one who has had the opportunity to examine the ambulance system in vogue in New York City, and to compare it with our own faulty and ineffectual police patrol system, the necessity of some more convenient and comfortable plan for the disposal of cases of emergency of various kinds, particularly cases of street accident, is but too manifest; but as there are many who are not familiar with the ambulance and its workings as illustrated by the system alluded to, a cursory survey of the subject may be of interest. The evils which the ambulance system is designed to combat have not been wholly abated by it, even in New York city, but the mere fact of their occasional unavoidable occurrence will serve to illustrate its necessity and manifold advantages. As an illustration of the numerous instances in which a well organized ambulance corps is a *sine qua non*, we will quote verbatim an item from a leading daily: "A few weeks ago a New York policeman arrested a man for drunkenness, who was afterward found to have been in a stupor caused by a fracture of his skull. Had the officer been fit for his position the man would have been taken to a hospital, instead of a police station. About the same time two Hartford policemen arrested Deacon Dewey, a respectable citizen of Granby, who was jolted from a load of tobacco in a street of the Connecticut capital. The good man's righteous indignation at the imputation of drunkenness has found expression in a suit for $6,000 damages for the false arrest. The other day a Brooklyn policeman arrested a respectable young married woman who stumbled in the street, and she was separated from her child and thrust into a cell on a charge of drunkenness. Her outraged husband did not discover her humiliating position until in the evening, and she was disgraced by the imposition of a fine before the magistrate released her. If she was sick, as she and her husband affirm, and not intoxicated, a jury would probably award her $10,000 damages for the indignity she has suffered. The people must be made to understand that it is a costly thing to allow an incompetent policeman to be intrusted with the power to make arrests, and when that fact is thoroughly understood at least 10 per cent. of the police of most cities will be dismissed." Now, it would seem that such strictures upon the police are entirely too severe, and it is just here that our own police patrol, albeit a very useful institution, which has been introduced as a remedy for such evils as accrue from the neglect of the injured, fails of its object. The police patrol system throws entirely too much responsibility upon the officers employed in its operation. We will take, for example, the case of a man who has been found lying in the street or in some out of the way locality. He is unconscious, and may or may not have received external injuries, or have taken liquor, the evidences of which are always sought for (and usually inferred to exist), and often the only thing manifest is that he is a stranger and unconscious. Now, let us consider for a moment what may be the matter with this man. Well, to begin with, he may be merely drunk, or he may have a condition of intoxication superadded to some serious injury; perhaps he has fallen upon his head while intoxicated, and received a fracture of the skull. He may be suffering from apoplexy, uræmic coma, or that condition of mental torpor so frequently noticed as a post-epileptic phenomenon. He may be under the influence of a narcotic poison, or perchance the soothing influence of the sand-bag; but in any case he is wholly or partially unconscious, and as before stated, he may have been drinking, the fumes of the liquor tending to confuse the case considerably. I will now ask my professional brethren if it is an easy matter to make a differential diagnosis under these circumstances? I imagine I hear the answer, "Very difficult sometimes outside of our text-books," and yet we expect our policemen to make the diagnosis. How natural it is for these officers to relegate the case to the domain of "drunk and disorderly!" They know little or nothing of the other conditions, and most obviously they are likely to see only that with which they are most familiar.

We will suppose that they take up this man, and their taking up is not apt to be very gentle if the odor of liquor be perceptible about the victim; place him in the bottom of the ordinary vehicle yclept "the patrol wagon," and jolt him off to the police station; place him in a cell and lock him up. In the morning the prisoner is found dead, and upon autopsy a clot of blood from a ruptured middle meningeal is found pressing upon the brain. Should these officers be indiscriminately censured for an accident due to their lack of surgical knowledge? I think not, for who among us but has made even greater mistakes? The illustration given is no fancy sketch, but is drawn from personal experience. In one instance occurring in the streets of New York City, I saw several officers roughly shaking a man whom I found upon examination to have just recovered from an epileptic fit. He had been drinking and the odor of the liquor led the unscientific minions of the law to assume that he was drunk, his mental torpor being supposed to be the obstinacy of drunkenness. How could these officers be expected to recognize the real condition of the man? There are, as I have stated, numerous conditions in which such mistakes might be made. We all know the difficulty of differentiating certain forms of coma. Hæmorrhage into the pons varolii and opium poisoning give very similar symptoms, and require careful consideration in diagnosticating the case, and if in such a case the patient should also happen to be drunk, the diagnosis would be doubly obscure. The author quoted from the daily press states that when certain facts are understood fully 10 per cent. of the police force will be dismissed; but looking at the matter from a medical standpoint, the whole police force would have to be discharged, if the ability to differentiate certain pathological states from "drunk and disorderly" was to be taken as a criterion of their capacity. But the municipality pays for policemen, and poorly enough too, and ought not to expect to get medical qualifications thrown in. It is fortunate for the officers that they are paid as policemen, and not as physicians, for the latter are not supposed to have any rights which the municipality is bound to respect. I might remark in this connection, that the policeman is expected to furnish a physician himself in all cases of emergency, particularly at the police stations, and to pay him out of his own pocket. If he fails to furnish a physician, he is liable to punishment, should any mishap occur through lack of medical attention. Should the physician send in his bill to the city, he will not receive the price of the paper it is written on in return. Now this system, or rather lack of system is all wrong. There is but one physician regularly employed by the city for such work, and it is obviously impossible for him to attend to it all. He is overworked and poorly paid. There should be in Chicago,as in New York city, a regular corps of police surgeons, one or two at least for each division of the city. These should have regular salaries, or should be fairly paid for each case, as under ordinary circumstances. In this way, all cases under police jurisdiction, requiring medical attention, could have it promptly and efficiently rendered, and the city would not be continually imposing a special tax upon both policemen and physicians. As a substitute for a corps of police surgeons, a provision might be made by which any physician could be called upon to care for an injured person, and could attend him with the security of being paid for his services.

Very often it would be desirable for a physician to accompany an injured person to the hospital or station to which he is removed by the patrol, but if he does so it is at his own expense, unless he can get some compensation from the patient, which is rarely the case. A regular system of ambulances under the management of the various hospitals is really a pressing want with us.

The ambulances of New York city are under the control of a number of hospitals, each one having its own line, with its corps of surgeons. Some of the most effective work is done by the ambulance attached to the Chambers Street Hospital, this institution being especially designed for cases of emergency, the best of the cases being retained by the staff, and the rest distributed among the other hospitals of the city. This institution uses the telephone from police headquarters. The signal, 5–5–4, is sent to the ambulance stable on Duane street. This calls up the driver, who is then told to drive to the place of the accident, where the ambulance surgeon is met, he having run to the scene while the horse was being harnessed. It takes about half a minute to prepare the ambulance and get under way. About sixteen calls a day are attended on the average. The district covered is not large, but three ambulances are required for the work. The Ninety-ninth Street Hospital ambulance service is not so complete as the others, but it covers more ground than any of them. The district runs from Sixty-first street to King's bridge, on the west side of the city, and from Seventy-fourth street to West Farms on the east side. The station houses in the district connect with the hospital by telegraph. When a gong at the back of the hospital is struck, a similar bell in the building notifies the surgeon. The horse is harnessed, the doors of the stable are thrown open at the same time, and the ambulance is away in two minutes. St. Vincent's Hospital runs two ambulances, and is the most perfect service in the city. One of these gets ready in fifteen seconds in the daytime and is rarely more than a minute getting under way at night. A call bell is by the doctor's bed. This awakens him, while at the same time a weight falls and lights the gas. He hastily dons his boots and trousers, and catching his coat from the rack on the way, rushes for the ambulance. While this is going on a bell strikes in the stable, a weight falls, opening the doors and unhitching the horse; the animal being trained runs under the harness, which is dropped and clasped in an instant, after the fashion of our hose-carts, and the machine is ready to start.

Bellevue Hospital runs four ambulances, and being a public institution, competes with all the others. The Presbyterian Hospital covers the territory controlled by the 59th and 88th street police stations. The ambulance gets under way in one minute after the alarm is sounded. The Roosevelt Hospital covers a district including the 20th and 22d wards. The distances traversed by some of these ambulances is, as may be noticed, sometimes very great. The spirit of competition between the various hospitals is very eager, each surgeon striving to capture the case for his own hospital. Sometimes three or four ambulances arrive on the scene of an accident at nearly the same time. It will be seen that the system is very thorough and efficient, and far superior to our police patrol. Instead of the patient being consigned to the care of police officers and hurried away over the rough pavement in an imperfectly equipped and uncomfortable wagon, he is placed in charge of skillful surgeons, and taken to the hospital as leisurely and comfortably as may be required. We have among us good hospitals, and in sufficient number to be all that is required, and I can see no valid reason why we should be behind the great metropolis in our hospital equipments, To be sure, our public institutions are run by politicians, while in New York the physicians have, at least, the dressing of wounds and the compounding of pills left to their own jndgment, but the public may not remain in darkness forever, and we may yet see a hospital millennium even in Chicago. Perhaps, however, a hospital millennium is already with us, for our Commissioners have established the fact that doctors do not need to know anatomy now-a-days, especially if they must disfigure the pauper form divine to obtain that knowledge. All that is required is that the medical lights shall be of good Republican or Democratic principles, as the case may be, and that each shall be a power in his ward. If a physician should be so unfortunate as to be connected with a college, and should be so audacious as to teach medicine and surgery, instead of politics, he is at once cast into outer darkness, but should he be able to discourse fluently upon the merits of the various brands of Sp. frumenti, and to control a certain amount of the political rabble, he is a fine fellow, and deserving of all consideration. Woe, woe to the unlucky wight who attempts to take advantage of our abundant material and advance our noble art! There is, I am happy to believe, a simple solution of the difficulty, and that is, the establishment of hospitals in connection with our medical colleges, a step in which Rush has already taken the initiative. I have made,I presume, quite a pronounced digression, but I am confident that it is apropos of the situation.

125 State St.

